# Indirect impact of SARS‐CoV‐2 pandemic on pregnancy and childbirth outcomes: A nine‐month long experience from a university center in Lombardy

**DOI:** 10.1002/ijgo.13990

**Published:** 2021-11-02

**Authors:** Sara Ornaghi, Simona Fumagalli, Chiara K. Guinea Montalvo, Greta Beretta, Francesca Invernizzi, Antonella Nespoli, Patrizia Vergani

**Affiliations:** ^1^ Department of Obstetrics and Gynecology Unit of Obstetrics MBBM Foundation Onlus at San Gerardo Hospital Monza Italy; ^2^ University of Milan‐Bicocca School of Medicine and Surgery Monza Italy

**Keywords:** care, childbirth, coronavirus disease 2019, pandemic, pregnancy, severe acute respiratory syndrome coronavirus 2

## Abstract

**Objective:**

To determine the impact on perinatal health of changes in social policies and obstetric care implemented to curb SARS‐CoV‐2 transmission. However, robust data on the topic are lacking since most of the studies has examined only the first few months of the outbreak.

**Methods:**

A retrospective analysis of prospectively collected data on uninfected and asymptomatically infected women giving birth between March and November 2020 and in the same time frame of 2019 at our tertiary care center in Lombardy, northern Italy. Perinatal outcomes were compared according to the year (2019 versus 2020) and to the trimester (March–May, June–August, September–November) of childbirth, corresponding to the three phases of the pandemic (first wave, deceleration, second wave) and covering a 9‐month period.

**Results:**

We identified increased rates of gestational diabetes mellitus, spontaneous preterm birth, and neuraxial analgesia in 2020 versus 2019, with different temporal distributions: gestational diabetes mellitus and spontaneous preterm birth were more prevalent during the deceleration and the second wave phase, whereas epidural analgesia was more prevalent during the first wave.

**Conclusion:**

By assessing a prolonged time frame of the pandemic, we show that pandemic‐related control measures, as applied in Lombardy, impacted relevant perinatal outcomes of women giving birth at our center.

## 1 INTRODUCTION

Since its start in March 2020, the severe acute respiratory syndrome coronavirus 2 (SARS‐CoV‐2) pandemic has substantially impacted perinatal health.^1^, ^2^, ^3^, ^4^, ^5^, ^6^, ^7^, ^8^, ^9^, ^10^

Northern Italy, and specifically the Lombardy region, was one of the hardest hit European areas at the beginning of the pandemic.^11^

On March 8, 2020, the Lombardy region issued a regionwide stay‐at‐home order, which was extended to all other Italian regions on March 11, 2020, and was maintained until May 18, 2020. Also, specific recommendations for maternity services were rapidly issued.^4^, ^7^, ^9^ A few tertiary‐care hospitals, including the research site, were chosen as referral centers for SARS‐CoV‐2‐infected obstetric patients.^4^ In‐person visits were maintained only if deemed necessary, and remotely delivered visits and childbirth preparation classes were implemented.^12^, ^13^ Skin‐to‐skin and birth companions were not allowed in SARS‐CoV‐2‐positive women till May and October 2020, respectively. Breastfeeding was permitted with the use of facemask and gloves in the case of confirmed infection. One support person was allowed during the postpartum stay only in SARS‐CoV‐2‐negative women for 2 h a day, whereas in‐hospital visits by family members were suspended.^14^

Although drafted to provide guidance and safety in the context of a constantly evolving situation and knowledge, these recommendations influenced crucial aspects of obstetric care.^1^, ^10^, ^15^, ^16^

Direct effects of the SARS‐CoV‐2 infection on pregnant women and their babies have been extensively investigated, with findings unanimously suggesting that moderate‐to‐severe symptomatic infection associates with adverse obstetric outcomes, such as preterm birth (PTB) and low birth weight.^17^, ^18^, ^19^, ^20^, ^21^, ^22^, ^23^, ^24^, ^25^, ^26^, ^27^ In turn, the literature examining whether the changes in social and obstetric care policies due to the pandemic have affected perinatal outcomes in uninfected and asymptomatically infected women is more controversial. Some studies have suggested that stay‐at‐home orders may have contributed to decreased rates of PTB,^28^, ^29^, ^30^, ^31^, ^32^, ^33^, ^34^, ^35^, ^36^, ^37^, ^38^ although available evidence is discordant.^39^, ^40^, ^41^, ^42^ Similarly, contrasting reports have been published regarding the risk of stillbirth.^38^, ^40^, ^41^, ^43^, ^44^, ^45^, ^46^ Of note, most of the research on the topic has focused only on the first few months of the pandemic, so has probably been unable to capture all the relevant outcomes and potential temporal trend differences.

The aim of this study was to investigate perinatal outcomes among uninfected and asymptomatically infected women who gave birth during a 9‐month time frame of the 2020 pandemic in a referral center for SARS‐CoV‐2 infection in obstetric patients in Lombardy, northern Italy, and to compare them to those of women in 2019.

We hypothesized that the modifications in women's life‐style, maternity services, and labor and birth practices due to the pandemic, as well as the protracted increased workload of midwives and obstetricians working in a referral center for SARS‐CoV‐2 infection, ultimately affected perinatal outcomes.

## 2 MATERIALS AND METHODS

This was a retrospective analysis of prospectively collected data including women giving birth between March 1 and November 30, 2020 and in the same time frame of 2019, at our tertiary‐care center.

Starting in March 2020, a comprehensive questionnaire was administered to all women at hospital admission.^47^ A targeted SARS‐CoV‐2 screening approach triggered by a positive questionnaire and based on reverse transcription polymerase chain reaction testing of nasopharyngeal swabs was used until April 8, 2020, when a universal viral screening policy was implemented. Healthcare personnel caring for suspected and confirmed SARS‐CoV‐2‐positive women in labor wore full personal protective equipment, including N95 facemask, gloves, goggles, and gown.

Data regarding general maternal characteristics, obstetric history, pregnancy course, and perinatal outcomes of women giving birth at our center are routinely recorded in a dedicated log book, which is periodically audited as part of our institutional quality improvement program aimed at safely reducing rates of obstetric interventions, including primary cesarean section (CS)^48^ and episiotomy.^49^

PTB was defined as any birth occurring before 37 weeks of pregnancy. It was categorized as spontaneous and medically indicated.

Gestational diabetes mellitus (GDM) was diagnosed by the one‐step approach using a 75‐g, 2‐hour oral glucose tolerance test as proposed by the International Association of Diabetes and Pregnancy Study Groups^50^ and as per institutional protocol since January 2011.

For the purpose of this study, only women with a laboratory‐confirmed, asymptomatic SARS‐CoV‐2 infection, as well as uninfected women, were included in the analyses. In turn, symptomatically infected women were excluded to avoid biases due to the known effects of symptomatic infection on the investigated outcomes.^17^, ^18^, ^19^, ^20^, ^21^, ^22^, ^23^, ^24^, ^25^, ^26^, ^27^ Asymptomatic infection was identified by a positive SARS‐CoV‐2 reverse transcription polymerase chain reaction in women with a negative admission questionnaire and no new onset of symptoms during the postpartum stay.

The study was approved by the Institutional Review Board of the University of Milan‐Bicocca (#15408/2020).

Assessment of normality was performed by means of Kolmogorov‐Smirnov test. Statistical analyses included χ^2^ test or Fisher exact test for categorical variables, and independent Student's *t* test for continuous variables.

We compared outcomes between women giving birth in March–November 2020 and March–November 2019 to account for seasonality.^51^ Outcomes of interest were also investigated according to the trimester of childbirth. We identified three trimesters (March to May, June to August, September to November) which correspond with the phases of the pandemic in Lombardy: first wave, deceleration, and second wave.^11^

A logistic regression model stratified by year of birth with 2019 group as referent was employed to estimate dose–response associations with perinatal outcomes, adjusted for potential confounding variables. Statistical significance was set at *P* < 0.05 (SPSS software, version 27; IBM, Armonk, NY, USA; graphpad prism software, version 7; GraphPad, San Diego, CA, USA).

## 3 RESULTS

**FIGURE 1:**
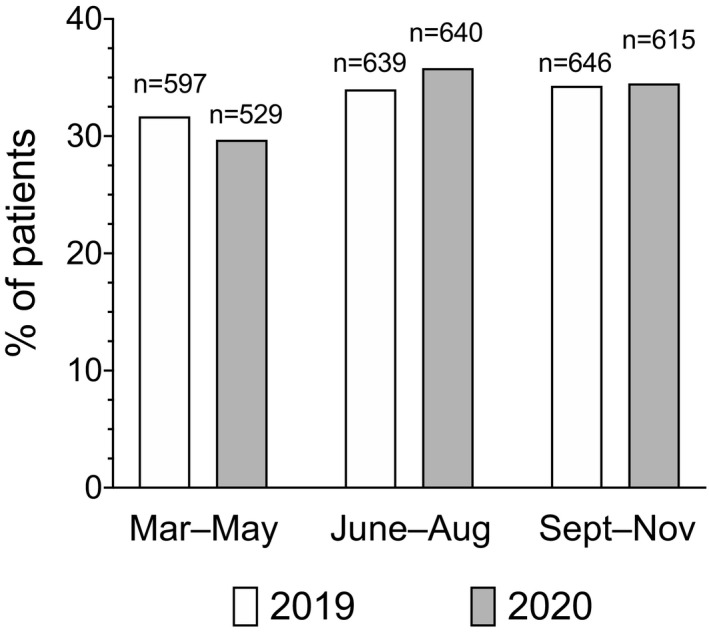
Temporal distribution of births at our center in 2019 and 2020

During the study period, there were 3666 births, 1882 in 2019 and 1784 in 2020. There were 113 multiple gestations (three were triplet gestations), for a cohort of 3782 fetuses. Figure 1 shows temporal distribution of births according to the pandemic phases in 2020 and the correspondent time frames in 2019.

Viral infection screening upon hospital admission for childbirth diagnosed 49 women as asymptomatically infected with SARS‐CoV‐2. During the 2020 study period, there were 35 additional women diagnosed as SARS‐CoV‐2 positive: 25 were mildly symptomatic, whereas 10 had pneumonia. These 35 women were not included in the analyses.

Women in 2020 were older, mostly more than 40 years old, and more frequently multiparas with a previous CS in their obstetric history compared with the women in 2019 (Table 1). No differences were identified regarding pregestational comorbidities or gestational complications, except for higher rates of GDM in 2020 (14.4% versus 11.3%, *P* = 0.005). Accordingly, polyhydramnios was more commonly diagnosed. There were 15 stillbirths, 8 (0.4%) in 2019 and 7 (0.4%) in 2020 (*P* = 1.000). None of the stillbirths diagnosed during the pandemic were in SARS‐CoV‐2‐positive, asymptomatic women.

**TABLE 1:** General and obstetric characteristics of women of the study cohort according to year of childbirth^a^

Variables	Overall study cohort *N* = 3666	2019 *N* = 1882	2020 *N* = 1784	*P* value
General characteristics				
Maternal age, year	32.8 ± 5.2	32.6 ± 5.3	33.1 ± 5.1	0.001
Maternal age >35 years	1123 (30.6)	565 (30.0)	558 (31.3)	0.410
Maternal age >40 years	245 (6.7)	108 (5.7)	137 (7.7)	0.011
BAME	442 (12.1)	213 (11.3)	229 (12.8)	0.171
Pregestational BMI	23.4 ± 4.6	23.2 ± 4.5	23.5 ± 4.7	0.069
Pregestational obesity^a^	324 (8.8)	152 (8.2)	172 (9.8)	0.092
Comorbidities				
Asthma	9 (0.2)	5 (0.3)	4 (0.2)	1.000
Chronic hypertension	66 (1.8)	30 (1.6)	36 (2.0)	0.385
Diabetes type I or II	15 (0.4)	5 (0.3)	10 (0.6)	0.199
Obstetric history and current pregnancy data				
Nulliparity^b^	1775 (48.4)	939 (49.9)	836 (46.9)	0.036
Previous cesarean section	449 (12.2)	212 (11.3)	237 (13.3)	0.035
Childbirth preparation classes^c^	1268 (34.6)	653 (34.8)	615 (34.5)	0.835
GDM	465 (12.7)	208 (11.1)	257 (14.4)	0.005
Cholestasis	109 (3.0)	48 (2.6)	61 (3.4)	0.144
HDP	153 (4.2)	79 (4.2)	74 (4.1)	1.000
Twin gestation	113 (3.1)	63 (3.3)	50 (2.8)	0.195
Oligohydramnios	94 (2.6)	42 (2.2)	52 (2.9)	0.060
Polyhydramnios	83 (2.3)	32 (1.7)	51 (2.9)	0.041
pPROM	107 (2.9)	48 (2.6)	59 (3.3)	0.103

Abbreviations: BAME, Black, Asian, and Minor Ethnicity; BMI, body mass index (calculated as weight in kilograms divided by the square of height in meters);GDM, gestational diabetes mellitus; HDP, hypertensive disorders of pregnancy, including gestational hypertension, pre‐eclampsia, pre‐eclampsia superimposed on chronic hypertension; pPROM, preterm prelabor rupture of membranes.

^a^ Values are presented as mean ± standard deviation or as number (percentage).

^b^ BMI >30.

^c^ Includes women with no previous birth >22 weeks of pregnancy.

^d^ Remotely delivered in 2020.

Overall, PTB before 37 weeks occurred in 266 (7.3%) women (Table 2). Assessment of PTB subtype showed increased rates of spontaneous PTB subsequent to preterm onset of labor among women giving birth in 2020 compared with 2019. Out of the 141 cases of PTB in 2020, only three occurred in SARS‐CoV‐2‐positive, asymptomatic women.

**TABLE 2:** Childbirth outcomes^a^

Variables	Overall study cohort *N* = 3666	2019 *N* = 1882	2020 *N* = 1784	*P* value
GA at birth, week	39.2 ± 2.3	39.1 ± 2.2	39.0 ± 2.3	0.334
PTB <37 weeks^b^	266 (7.3)	125 (6.7)	141 (7.9)	0.143
PTB 32–36^+6^ weeks^b^	206 (5.6)	95 (5.1)	111 (6.2)	0.132
Spontaneous PTB^b^	169 (4.6)	73 (3.9)	96 (5.4)	0.033
Labor	3220 (87.8)	1669 (88.7)	1551 (86.9)	0.117
Induced	902 (24.6)	459 (27.5)	443 (28.6)	0.505
Neuraxial analgesia in labor	1036 (28.3)	488 (25.9)	548 (30.7)	0.001
Among women with no previous vaginal birth	830/2127 (22.6)	393/1109 (35.4)	437/1018 (42.9)	<0.001
Oxytocin in labor	626 (17.1)	332 (17.6)	294 (16.5)	0.357
Cesarean section	752 (20.5)	382 (20.3)	370 (20.7)	0.744
Pre‐labor	368 (10.0)	183 (47.9)	185 (50.0)	0.545
Robson class 1 cesarean section^c^	272 (7.4)	154 (8.2)	118 (6.6)	0.040
Operative vaginal birth	121 (3.3)	56 (3.0)	65 (3.6)	0.268
Postpartum hemorrhage				
>1000 ml	168 (4.6)	93 (4.9)	75 (4.2)	0.305
>2000 ml	20 (0.5)	14 (0.7)	6 (0.3)	0.117
Episiotomy	303 (8.3)	183 (9.7)	120 (6.7)	0.001
Perineal tears III–IV degree	36 (1.0)	16 (0.9)	20 (1.1)	0.503
Breastfeeding^d^	3537 (96.5)	1822 (96.8)	1715 (96.1)	0.151

Abbreviations: CS, cesarean section; GA, gestational age; PTB, preterm birth.

^a^ Values are presented as mean ± standard deviation or as number (percentage).

^b^ Includes only pregnancies with birth after 22 weeks or pregnancy and no stillbirth.

^c^ Includes women with no previous birth after 22 weeks of pregnancy, at term, with a singleton pregnancy, a fetus in vertex presentation, and a spontaneous onset of labor.

^d^ Assessed at hospital discharge.

Approximately one‐third of women used neuraxial analgesia in labor during the 2 year‐study period, with higher rates in 2020 versus 2019. An even more substantial increase was identified in 2020 when analysis was restricted to only those women with no previous vaginal birth.

Globally, frequencies of abdominal and vaginal operative birth were not affected by the pandemic; however, we observed a reduction in CS rate from 2019 to 2020 among women in Robson class 1 (8.2% versus 6.6%, *P* = 0.04). Similarly, episiotomy rates displayed a substantial decline (9.7% versus 6.7%, *P* = 0.001).

No differences were identified regarding short‐term neonatal outcomes, including birthweight (mean ± standard deviation: 3170.5 ± 588.6 g in 2019 versus 3187.1 ± 596.8 g in 2020, *P* = 0.388) and Apgar score at 5 min (9.8 ± 0.6 in 2019 versus 9.8 ± 0.7 in 2020, *P* = 0.153). Overall, breastfeeding at hospital discharge was present in 3537 (96.5%) of women, with similar rates between 2019 and 2020.

All findings of the univariate analysis, except for polyhydramnios, were confirmed by the logistic regression model adjusted for potential confounding factors (Table 3).

**TABLE 3:** Logistic regression model of perinatal outcomes of interest

Variables	2019	2020		2020	
		OR	95% CI	aOR	95% CI
GDM^a^	Ref.	1.33	1.09–1.61	1.28	1.05–1.56
Polyhydramnios^b^	Ref.	1.37	1.12–1.84	1.04	0.88–1.27
Spontaneous PTB^c^	Ref.	1.42	1.04–1.93	1.43	1.05–1.95
Epidural analgesia^d^	Ref.	1.27	1.10–1.46	1.52	1.04–2.31
Robson class 1 CS^e^	Ref.	0.79	0.59–0.96	0.63	0.51–0.79
Episiotomy^d^	Ref.	0.67	0.53–0.85	0.69	0.54–0.88

Abbreviations: aOR, adjusted odds ratio; CI, confidence interval; CS, cesarean section; GDM, gestational diabetes mellitus; OR, odds ratio; PTB, preterm birth; SARS, CoV‐2, severe acute respiratory syndrome coronavirus 2.

^a^ Adjusted for maternal age over 40 years, pregestational obesity, and nulliparity.

^b^ Adjusted for maternal age over 40 years, pregestational obesity, nulliparity, and GDM.

^c^ Adjusted for maternal age over 40 years, pregestational obesity, nulliparity, SARS‐CoV‐2 infection, GDM, and polyhydramnios.

^d^ Adjusted for maternal age over 40 years, pregestational obesity, previous cesarean section, nulliparity, SARS‐CoV‐2 infection, GDM, polyhydramnios, and spontaneous PTB.

^e^ Adjusted for maternal age over 40 years, pregestational obesity, SARS‐CoV‐2 infection, GDM, polyhydramnios, epidural analgesia, and spontaneous PTB.

**FIGURE 2:**
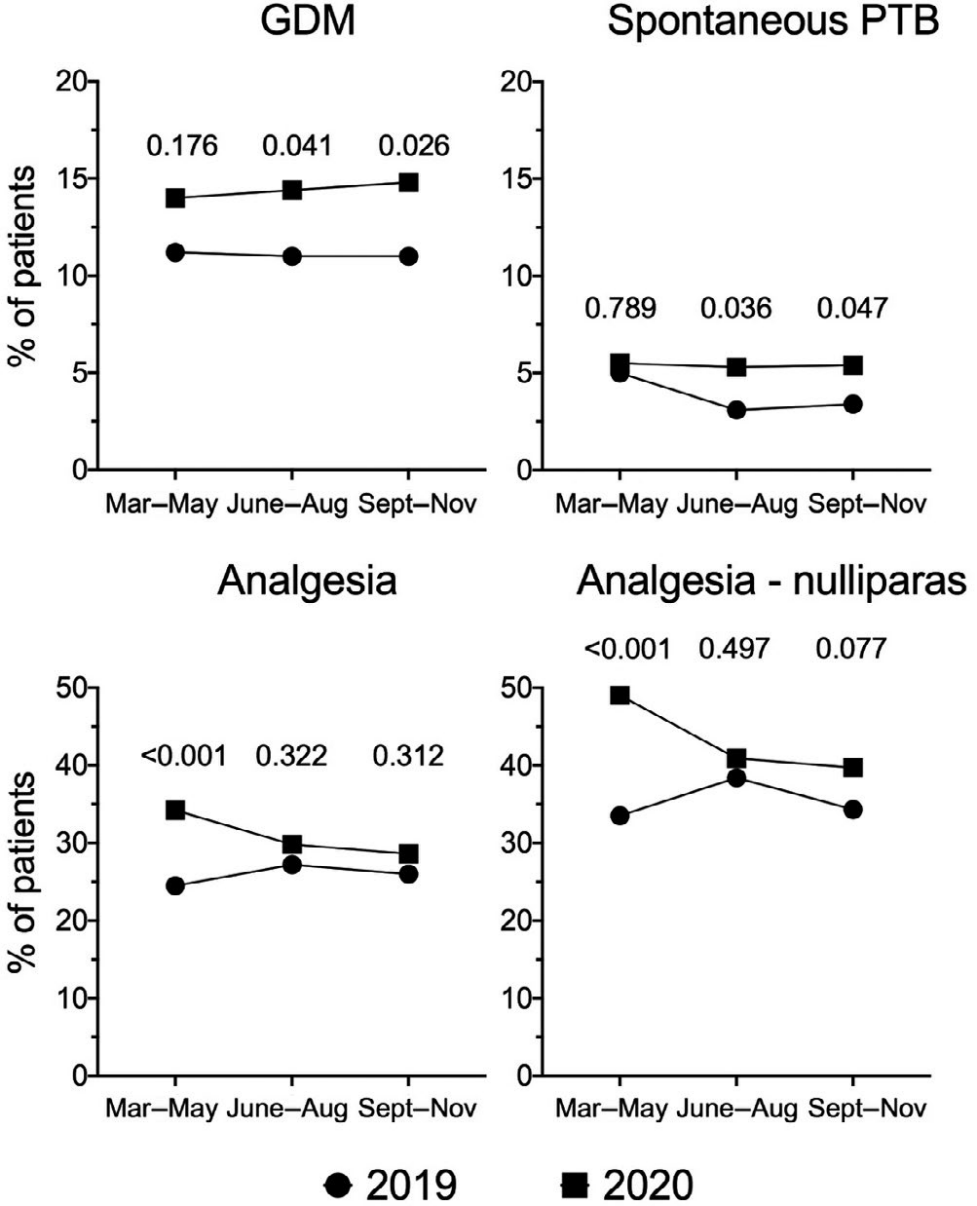
Perinatal outcomes according to the trimester of birth in 2019 and 2020. GDM, gestational diabetes mellitus; PTB, preterm birth (birth before 37 weeks). Analgesia—nulliparas: use of neuraxial analgesia in labor among women with no previous vaginal birth after 22 weeks of pregnancy

Analyses according to the trimester of birth identified temporal differences in the assessed perinatal outcomes, with increased rates of GDM and spontaneous PTB during the deceleration and the second wave phase of the pandemic, and of epidural analgesia during the first pandemic wave (Figure 2).

A sensitivity analysis including only SARS‐CoV‐2‐negative women showed results similar to the analyses of the overall study population (see Tables S1–S3).

## 4 DISCUSSION

Our data support the hypothesis that modifications in social policies and in labor and birth practices abruptly introduced to contain viral spread at the beginning of the outbreak influenced perinatal outcomes of uninfected and asymptomatically infected women giving birth in a referral center for SARS‐CoV‐2 infection in Lombardy, northern Italy, over a prolonged period of time.

We observed increased rates of GDM, spontaneous PTB, and use of neuraxial analgesia in labor in 2020 versus 2019. These outcomes displayed a different temporal distribution, with GDM and spontaneous PTB being more prevalent during the deceleration and the second wave phase and epidural analgesia being more prevalent during the first wave phase. In turn, we identified a substantial reduction in the 2020 rates of obstetric interventions, including CS among women in Robson class 1 and episiotomy.

As governments and hospitals worldwide continue to work to effectively protect women and their healthcare providers from SARS‐CoV‐2 infection, it remains important to determine to what extent swift modifications in social and obstetric care policies designed to curb viral transmission impact perinatal health.^52^ Although much has been written on this topic, there are few actual data because most of the research work has assessed short time periods limited to the first few months of the pandemic.^15^, ^29^, ^30^, ^31^, ^33^, ^36^, ^39^, ^41^, ^46^, ^53^, ^54^ Literature published quickly after new policy implementation is unlikely to capture all relevant outcomes. Also, policies have substantially varied over time. As a result, assessment of longer time frames, as we did in our study, is crucial to obtain more informative data.

We identified a higher risk of GDM in the 2020 versus 2019 cohort of pregnant women, with a substantial increase during the deceleration (June–August) and the second wave (September–November) phases.

This is in line with a recent report from Israel.^53^ Interestingly, a survey among Spanish pregnant women has identified a decrease in levels of physical activity during implementation of lockdown measures.^55^ Regular exercise during pregnancy is known to reduce the odds of obstetric complications, including GDM.^56^, ^57^, ^58^, ^59^ Strict home confinement was mandatory in the Lombardy region from March 8 till May 18, 2021. This may have affected levels of exercise in pregnant women, especially in those in their first and early second trimesters, thus ultimately favoring GDM.

Contrasting data have been published regarding the rates of spontaneous PTB during the first months of the pandemic compared with previous years, suggesting a protective,^28^, ^29^, ^30^, ^31^, ^32^, ^33^, ^34^, ^35^, ^36^, ^37^, ^38^ detrimental,^40^ or non‐existent effect^38^, ^39^, ^41^, ^42^ of strict stay‐at‐home orders on this obstetric complication.

By assessing a 9‐month time frame encompassing three sequential phases of the pandemic, we observed similar rates of spontaneous PTB during the first wave (March–May), when lockdown measures were in place, and increased rates after such measures were discontinued, compared with the same months of 2019. Reduced in‐person antenatal visits may have contributed to these findings.^40^, ^41^ Also, high levels of prenatal stress and anxiety, which have been reported in pregnant women exposed to the pandemic‐related lockdown^35^, ^60^, ^61^, ^62^, ^63^, ^64^, ^65^, ^66^, ^67^, ^68^, ^69^, ^70^, ^71^ and are risk factors for PTB,^72^, ^73^ might have played a role. Another possible explanation includes change in the referral patterns with more high‐risk women referred to our university center.^41^, ^53^, ^74^ In addition, the effect of population heterogeneity cannot be excluded, as our 2019 spontaneous PTB rates were lower than those of other published cohorts reporting a decline likely related to a beneficial role of stay‐at‐home orders,^28^, ^29^, ^30^, ^31^, ^32^, ^33^, ^34^, ^35^, ^36^, ^37^, ^38^ suggesting a potential divergent influence of such orders on different pregnant populations.

Withdrawal of epidural services has been reported in some of the most affected Italian regions.^52^ Although being a referral center, we were able not only to guarantee neuraxial analgesia in labor as we did in 2019 but also to face an increased request for this during the first wave. Substantial levels of stress and anxiety, due to the limited knowledge of the effects of the virus on the mother and her fetus and the radical changes swiftly implemented in labor and birth care^35^, ^60^, ^61^, ^62^, ^63^, ^64^, ^65^, ^66^, ^67^, ^68^, ^69^, ^70^, ^71^ might explain this increase and its temporal trend.

Regarding obstetric interventions, our data showed a reduction in the 2020 rates of CS among women in Robson class 1 as well as of episiotomy, whereas there were no differences regarding the rates of labor induction. These results are in contrast with a recent Italian report identifying higher rates of induced labor and CS on maternal request during the first months of the pandemic.^54^ As quality improvement initiatives have already been proved effective in ameliorating birth outcomes,^75^, ^76^, ^77^ we believe that our findings are the result of the constant effort to safely improve childbirth care at our Institution.

The strength of this study is the thorough evaluation of the impact of rapidly implemented modifications in social policies and obstetric care pathways, focused on reducing SARS‐CoV‐2 transmission, over a prolonged period of time. Assessment of three sequential phases of the pandemic, i.e. the first wave, the deceleration phase, and the second wave, allowed us to appreciate temporal trends of the affected outcomes, revealing insightful differences that would not have been clear with a shorter time‐frame evaluation, as recently suggested.^42^, ^78^ In addition, the use of a comparable period in 2019 limited potential biases related to seasonality of obstetric complications.^51^ Importantly, evaluation of several variables allowed us to adjust the analyses for confounding factors.

This study is not without limitations. First, the retrospective nature of the design prohibited us from establishing the causality of the association. Second, it is possible that this study was underpowered to assess differences in less common but severe adverse outcomes, including stillbirth. Third, our findings are from a single SARS‐CoV‐2 referral center, and may not be generalizable to different settings. Fourth, it is possible that some asymptomatically infected women might have been missed before universal SARS‐CoV‐2 screening was implemented on April 8, 2020; however, this is unlikely to have biased our findings because of the short time frame of universal testing unavailability compared with the entire study period, as well as of the high accuracy of the admission questionnaire guiding the targeted viral screening.^47^ Finally, we did not measure levels of stress and anxiety or of physical exercise among our cohort of pregnant women.

In conclusion, we continue to live through this pandemic. Our research work, encompassing a 9‐month time frame of the outbreak, provides a unique insight into its indirect effects on perinatal health. Importantly, our data suggest that these effects may vary according to the trimester of pregnancy in which women are when strict stay‐at‐home orders and changes in labor and birth care pathways were implemented.

Ongoing review of maternity statistics is warranted to remain vigilant for newly developing trends, in order to provide up‐to‐date evidence to optimally guide our service organization. In addition, interventions aiming to counteract the observed adverse perinatal outcomes, such as online programs to support healthy lifestyle habits and mental well‐being in pregnancy, should be promoted; this would be of utmost importance in the face of possible new situations of individual confinement.

## CONFLICT OF INTEREST

The authors have no conflicts of interest.

## AUTHOR CONTRIBUTIONS

SO contributed to the design, planning, and conduct of the study, performed the data analysis, wrote the manuscript, and contributed to its review. SF, AN, and PV contributed to the design, planning, and conduct of the study, and to manuscript review. CKGM, GB and FI contributed to the conduct of the study and to manuscript review. AN: design, planning, and conduct of the study, manuscript reviewing.

## Supporting information

Table S1‐3
