# Supplementary material for: Indirect impact of SARS‐CoV‐2 pandemic on pregnancy and childbirth outcomes: A nine‐month long experience from a university center in Lombardy
Source: Int J Gynaecol Obstet. 2021 Nov 2;156(3):466–74. doi: 10.1002/ijgo.13990 (PMC9087530; doi:10.1002/ijgo.13990)
Supplement: Supplementary file 1 — Table S1‐3 [file IJGO-156-466-s001.docx]

**Table S1. General and obstetric characteristics of not-infected women according to year of childbirth.**

| **Variables** | **2019**  **N=1882** | **2020**  **N=1735** | **p-value** |
| --- | --- | --- | --- |
| **General characteristics** | | | |
| Maternal age (years) | 32.6±5.3 | 33.2±5.1 | <0.001 |
| Maternal age >35 years | 565 (30.0) | 544 (31.4) | 0.387 |
| Maternal age >40 years | 108 (5.7) | 135 (7.8) | 0.017 |
| BAME | 213 (11.3) | 213 (12.3) | 0.380 |
| Pregestational BMI (kg/m^2^) | 23.2±4.5 | 23.5±4.7 | 0.126 |
| Pregestational obesity ^1^ | 152 (8.2) | 163 (9.5) | 0.157 |
| **Comorbidities** | | | |
| Asthma | 5 (0.3) | 4 (0.2) | 1.000 |
| Chronic hypertension | 30 (1.6) | 35 (2.0) | 0.381 |
| Diabetes type I or II | 5 (0.3) | 10 (0.6) | 0.196 |
| **Obstetric history and current pregnancy data** | | | |
| Nulliparity ^2^ | 939 (49.9) | 817 (47.1) | 0.049 |
| Previous cesarean section | 212 (11.3) | 229 (13.2) | 0.042 |
| Childbirth preparation classes ^3^ | 653 (34.8) | 604 (34.8) | 1.000 |
| GDM | 208 (11.1) | 246 (14.2) | 0.009 |
| Cholestasis | 48 (2.6) | 60 (3.5) | 0.118 |
| HDP | 79 (4.2) | 72 (4.1) | 1.000 |
| Twin gestation | 63 (3.3) | 48 (2.8) | 0.335 |
| Oligohydramnios | 42 (2.2) | 46 (2.7) | 0.225 |
| Polyhydramnios | 32 (1.7) | 48 (2.8) | 0.046 |
| pPROM | 48 (2.6) | 57 (3.3) | 0.198 |

BAME, Black, Asian, and Minor Ethnicity; GDM, gestational diabetes mellitus; HDP, hypertensive disorders of pregnancy, including gestational hypertension, preeclampsia, preeclampsia superimposed to chronic hypertension; pPROM, preterm premature rupture of membranes.

^1^ Body Mass Index > 30 Kg/m^2^.

^2^ Includes women with no previous birth > 22 weeks’ gestation.

^3^ Remotely delivered in 2020.

**Table S2. Childbirth outcomes among not-infected women giving birth in 2019 and 2020.**

| **Variables** | **2019**  **N=1882** | **2020**  **N=1735** | **p-value** |
| --- | --- | --- | --- |
| GA at birth (weeks) | 39.1±2.2 | 39.0±2.3 | 0.331 |
| Preterm birth <37 weeks ^1^ | 125 (6.7) | 138 (8.0) | 0.140 |
| Preterm birth 32 – 36^6/7^ weeks ^1^ | 95 (5.1) | 108 (6.3) | 0.129 |
| Spontaneous preterm birth ^1^ | 73 (3.9) | 93 (5.4) | 0.032 |
| Labor   - Induced | 1669 (88.7)  459 (27.5) | 1508 (86.9)  422 (28.0) | 0.114  0.781 |
| Neuraxial analgesia in labor   - Among women with no previous vaginal birth | 488 (25.9)  393/1109 (35.4) | 525 (30.3)  421/992 (42.4) | 0.004  0.001 |
| Oxytocin in labor | 332 (17.6) | 284 (16.4) | 0.330 |
| Cesarean section   - Pre-labor | 382 (20.3)  183 (47.9) | 354 (20.4)  181 (51.1) | 0.967  0.507 |
| Robson class 1 cesarean section ^2^ | 154 (8.2) | 112 (6.5) | 0.027 |
| Operative vaginal birth | 56 (3.0) | 65 (3.6) | 0.268 |
| Postpartum hemorrhage   - >1000 mL - >2000 mL | 93 (4.9)  14 (0.7) | 74 (4.3)  6 (0.3) | 0.342  0.120 |
| Episiotomy | 183 (9.7) | 117 (6.7) | 0.001 |
| Perineal tears III-IV degree | 16 (0.9) | 20 (1.2) | 0.404 |
| Breastfeeding ^3^ | 1822 (96.8) | 1709 (98.5) | 0.074 |

GA, gestational age; CS, cesarean section.

^1^ Includes only pregnancies with birth > 22 weeks’ gestation and no stillbirth.

^2^ Includes women with no previous birth > 22 weeks’ gestation, at term, with a singleton pregnancy, a fetus in vertex presentation, and a spontaneous onset of labor.

^3^ Assessed at hospital discharge.

**Table S3. Logistic regression model of perinatal outcomes of interest among not-infected women.**

| **Variables** | **2019** | **2020** | | **2020** | |
| --- | --- | --- | --- | --- | --- |
|  |  | **OR** | **95% CI** | **aOR** | **95% CI** |
| GDM ^1^ | Ref. | 1.30 | 1.07-1.58 | 1.26 | 1.03-1.54 |
| Polyhydramnios ^2^ | Ref. | 1.28 | 1.09-1.54 | 1.07 | 0.93-1.34 |
| Spontaneous PTB ^3^ | Ref. | 1.42 | 1.04-1.94 | 1.44 | 1.06-1.98 |
| Epidural analgesia ^4^ | Ref. | 1.24 | 1.07-1.43 | 1.26 | 1.08-1.48 |
| Robson class 1 CS ^5^ | Ref. | 0.77 | 0.60-0.98 | 0.74 | 0.57-0.95 |
| Episiotomy ^4^ | Ref. | 0.67 | 0.53-0.86 | 0.68 | 0.53-0.87 |

GDM, gestational diabetes mellitus; PTB, preterm birth, CS, caesarean section.

^1^ Adjusted for maternal age >40 years, pregestational obesity, and nulliparity.

^2^ Adjusted for maternal age >40 years, pregestational obesity, nulliparity, and GDM.

^3^ Adjusted for maternal age >40 years, pregestational obesity, nulliparity, GDM, and polyhydramnios.

^4^ Adjusted for maternal age >40 years, pregestational obesity, previous caesarean section, nulliparity, GDM, polyhydramnios, and spontaneous PTB.

^5^ Adjusted for maternal age >40 years, pregestational obesity, GDM, polyhydramnios, epidural analgesia, and spontaneous PTB.
